# Hexanuclear copper(II) complex of 2-hy­droxy-*N*,*N*′-bis­[1-(2-hy­droxy­phen­yl)ethyl­idene]propane-1,3-di­amine incorporating an open-cubane core

**DOI:** 10.1107/S2056989021005570

**Published:** 2021-06-11

**Authors:** Momath Kébé, Ibrahima Elhadji Thiam, Mouhamadou Moustapha Sow, Ousmane Diouf, Aliou Hamady Barry, Abdou Salam Sall, Pascal Retailleau, Mohamed Gaye

**Affiliations:** a Département de Chimie, Faculté des Sciences et Techniques, Université Cheik Anta Diop, Dakar, Senegal; b Département de Chimie, Faculté des Sciences et Techniques, Université Alioune Diop, Bambey, Senegal; c Département de Chimie, Faculté des Sciences et Techniques, Université Nouakchott Al Aasriya, Nouakchott, Mauritania; d Substances Naturelles, CNRS UPR 2301, Université Paris-Sud, Université, Paris-Saclay, 1 av. de la Terrasse, 91198 Gif-sur-Yvette, France

**Keywords:** crystal structure, 1-(2-hy­droxy­phen­yl)ethanone, 1,3-di­amino­propan-2-ol, open-cubane, hydrazone

## Abstract

The title mol­ecular structure, namely, di­aqua­tris­(μ_3_-1,3-bis­{[1-(2-oxidophen­yl)ethyl­idene]amino}­propan-2-olato)-μ_3_-hydroxido-dinitrato­hexa­copper(II) ethanol tris­olvate, [Cu_6_(C_19_H_19_N_2_O_3_)_3_(NO_3_)_2_(OH)(H_2_O)_2_]·3C_2_H_5_OH, corres­ponds to a non-symmetric hexa­nuclear copper complex. The complex exhibits one core in which three Cu^II^ metal centres are mutually inter­connected, two by two, *via* three phenolato oxygen anions acting in a μ_2_-mode. These three copper cations are inter­connected in a μ_3_-mode by one hydroxyl group. An open-cube structure is generated in which each of the Cu^II^ cations of the three CuO_4_N units is connected by two μ_2_-O anions from phenolate groups and one μ_3_-O atom from a hy­droxy anion. Each of the three penta­coordinated Cu^II^ cations situated in the open-cube unit has a distorted NO_4_ square-pyramidal environment. Each of these three Cu^II^ centres is inter­connected with another Cu^II^ cation *via* one enolate O atom in μ_2_-mode, yielding one CuNO_4_ unit and two CuNO_3_ units. The penta­coordinated Cu^II^ atom has a distorted square-pyramidal environment while the two tetra­coordinated copper(II) cations are situated in a square-planar environment. A series of intra­molecular O—H⋯O hydrogen bonds are observed. In the crystal, the units are connected two by two by inter­molecular C—H⋯O and O—H⋯O hydrogen bonds, thus forming sheets parallel to the *ac* plane.

## Abstract 2

In the title Schiff base hexa­nuclear copper(II) complex, two discrete environments are present in the structure: CuNO_4_ and CuNO_3_. Four copper(II) cations are situated in a distorted square-pyramidal environment, while two copper(II) cations are located in a slightly square-planar geometry. Three of the copper(II) cations occupy three vertices of an open cubane Cu_3_O_4_.

## Chemical context   

The coordination chemistry of penta­dentate ligands has been studied extensively. That their structures present symmetrical or asymmetrical pendant arms and bear donor atoms is an asset widely exploited in coordination chemistry. The presence of donor sites on aliphatic or aromatic arms has made it possible to prepare a wide variety of compounds with various structures and inter­esting physical and chemical properties. 1,3-Di­amino­propan-2-ol, which has three donor sites, is a good precursor for the synthesis of ligands with several cavities that can act as chelating agents and/or as bridging ligands (Song *et al.*, 2004 ▸; Shit *et al.*, 2013 ▸). These types of ligands can generate high nuclearity complexes with original structures. Indeed, ligands rich in hydroxyl groups and containing other donor sites such as nitro­gen are used to prepare complexes with very diverse structures (Gungor & Kara, 2015 ▸; Dutta *et al.*, 2020 ▸; Shit *et al.*, 2013 ▸; Sarı *et al.*, 2006 ▸). Several synthetic strategies have been developed to control the nuclearity and lead to specific applications in mol­ecular magnetism (Popov *et al.*, 2012 ▸; Mikuriya *et al.*, 2018 ▸), mol­ecular biology (Grundmeier & Dau, 2012 ▸), electrochemistry (Musie *et al.*, 2003 ▸) and catalysis (Gamez *et al.*, 2001 ▸). The self-assembly synthetic strategy involving transition-metal cations and multidentate ligands has been widely used by coordination chemists, as a result of the wide variety of fascinating structures with the presence of multiple metal centres. The high nuclearity of these complexes and the inter­actions that can take place between metal cations has increased their inter­est to chemists (Bonanno *et al.*, 2018 ▸; Yang *et al.*, 2014 ▸; Haldar *et al.*, 2019 ▸).
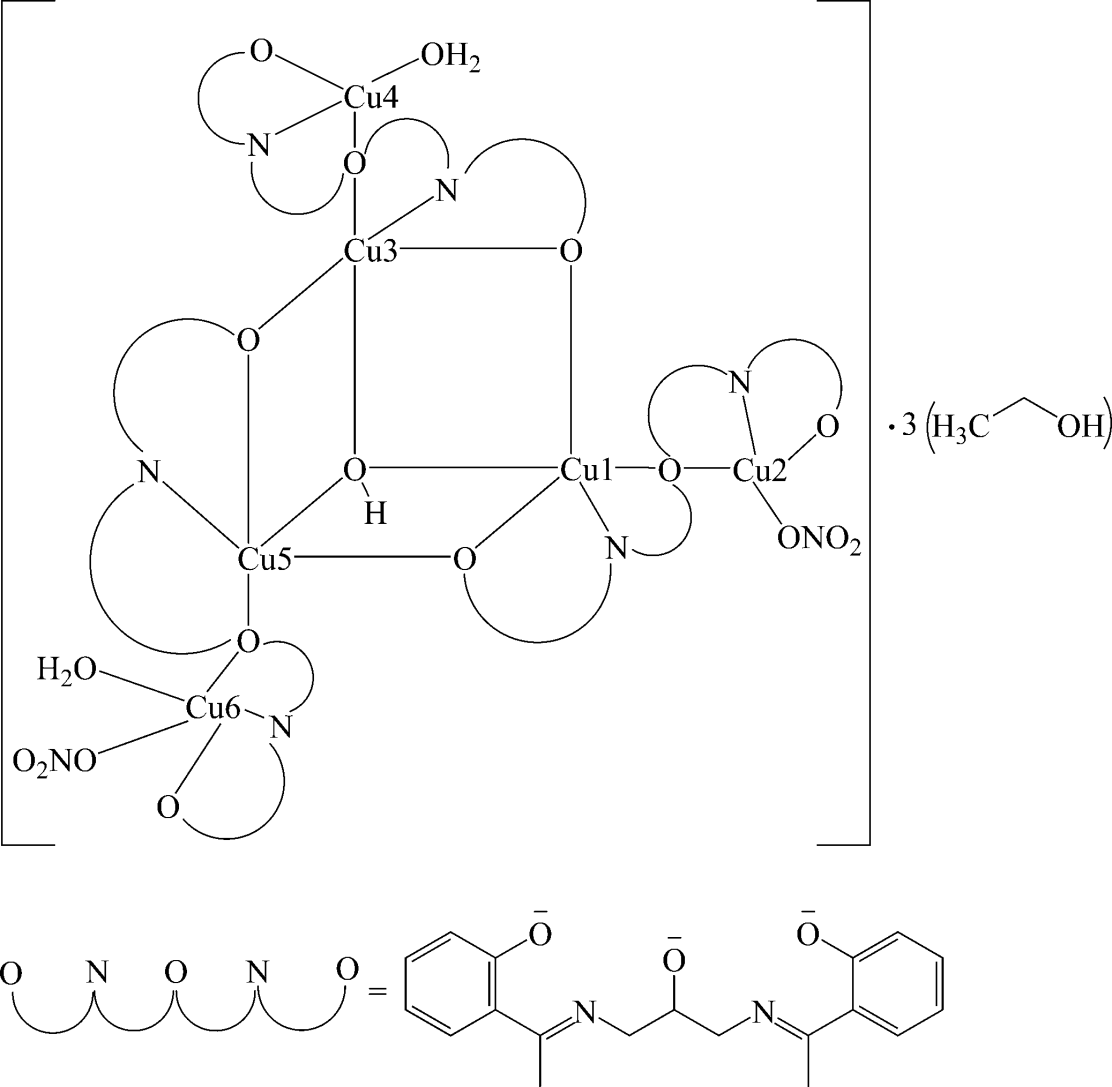


In a continuation of our work on multidentate Schiff base complexes (Sall *et al.*, 2019 ▸; Sarr *et al.*, 2018*a* ▸,*b* ▸; Mamour *et al.*, 2018 ▸), we have explored the possibility of preparing high nuclearity complexes using a Schiff base rich in hydroxyl groups. From 1,3-di­amino­propan-2-ol and 1-(2-hy­droxy­phen­yl)ethanone, we obtained a ligand containing three hydroxyl groups. The reaction of this ligand with copper nitrate resulted in the hexa­nuclear title complex, whose structure presents an open cube involving three of the six copper cations.

## Structural commentary   

**Figure 1:**
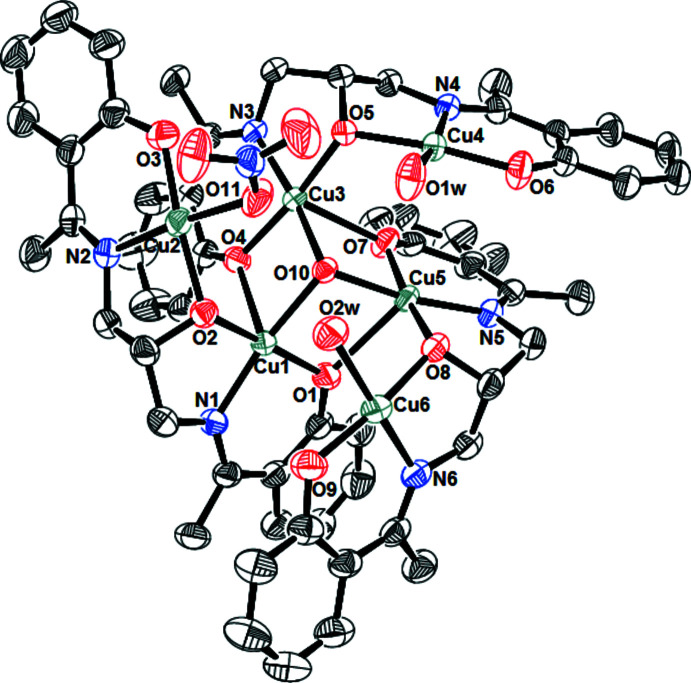
A view of the title compound, showing partial atom-numbering scheme. Displacement ellipsoids are plotted at the 30% probability level. H atoms and solvent molecules and atom labels for C atoms have been omitted for clarity.

The reaction of 1-(2-hy­droxy­phen­yl)ethanone and 1,3-di­amino­propan-2-ol in a 2:1 ratio in ethanol yielded the ligand *N*,*N*′-bis­{[1-(2-hy­droxy­phen­yl)ethyl­idene]}-2-hy­droxy­pro­pane-1,3-di­amine (H_3_ *L*). The reaction of ligand H_3_ *L* with copper nitrate yielded a complex in which the ligand reacted in tri-deprotonated form as *L* ^3−^. The coordination complex is formulated as [Cu_6_ *L*_3_(NO_3_)_2_(OH)(H_2_O)_2_]·3(EtOH) (I) (Fig. 1 ▸). In this hexa­nuclear open-cubane complex, each of the tri-deprotonated ligand acts as a bridge linking one copper(II) cation to two neighbouring Cu^II^ cations. The two imino nitro­gen atoms of the ligand are coordinated to two different Cu cations. One of the phenolato O atoms bridges two copper cations, while the second phenolato O atom is coordinated to a third copper cation. The third copper cation is bridged to the central copper cation *via* the enolato oxygen anion. The tri-deprotonated ligand coordinates in a hepta­dentate mode (μ_2_-O_phenolate_, *η* ^1^-N_imino_, μ_2_-O_enolato_, *η* ^1^-N_imino_, *η* ^1^-O_phenolato_), thus forming four fused chelate rings (two five-membered and two six-membered). Two discrete environments are observed in the structure: CuNO_4_ and CuNO_3_. The coordination environments for Cu1, Cu3, Cu5 and Cu6 are best described as square-pyramidal, as shown by the Addison τ parameter calculated from the largest angles (Table 1 ▸) around Cu1, Cu3, Cu5 and Cu6: τ = 0.045 (Cu1), τ = 0.007 (Cu3), τ = 0.010 (Cu5), τ = 0.040 (Cu6), (τ = 0 or 1 for perfect square-pyramidal and trigonal–bipyramidal geometries respectively). For Cu6, the basal plane is occupied by one phenolato oxygen anion, one enolate oxygen anion, one water O atom and one azomethine nitro­gen atom, the apical position being occupied by an anion oxygen of an unidentate nitrate group. The donor atoms (O8, N6, O9, O2*W*) of the basal coordination plane are almost coplanar and the Cu6 cation is displaced toward the apical atom (O201) by 0.0963 (9) Å. The *cissoid* angles are in the range 86.12 (9)–94.66 (9)° while the *transoid* angles are 171.23 (9) and 174.18 (9)°. In the basal plane, the Cu6—N6 [1.942 (2) Å] and the Cu6—O_ligand_ distances [1.935 (2) and 1.863 (2) Å] are shorter than the distance of Cu6—O2*W* [2.028 (2) Å]. The distance between the copper and the nitrato oxygen anion [Cu6—O14*B* = 2.45 (2) Å] in the apical position is longer than the distances to the atoms in the equatorial plane because of Jahn–Teller distortion, which is typical for copper(II) *d* ^9^ atoms (Monfared *et al.*, 2009 ▸). This distance is in accordance with reported values for nitrato square-pyramidal copper complexes (Noor *et al.*, 2015 ▸).

For Cu1, Cu3 and Cu5, which are situated on the vertices of the Cu_3_O_4_ open cube, the basal planes are occupied by one imino nitro­gen atom, one phenolate oxygen anion, one enolato oxygen anion from the same ligand mol­ecule and the O atom of the hy­droxy oxygen anion that connects the three copper cations. The copper cations situated on the corners of the open cube are connected by two μ_2_-O_phenolato_ and one μ_3_-O_hy­droxy_ atoms. In each case, the apical position is occupied by one phenolate oxygen anion from another ligand. The donor atoms of the basal coordination planes of Cu1, Cu3 and Cu5 centres are situated almost in the same plane and the copper cations are displaced from the corresponding apical positions [−0.1462 (8) Å for Cu1, −0.1253 (8) Å for Cu3 and 0.1122 (8) Å for Cu5). The open cube, defined as cube missing one corner, is distorted, as shown by the Cu—O—Cu [93.56 (8)–106.62 (9)°] and O—Cu—O [72.34 (7)–86.17 (8)°] angles, which deviate severely from the ideal value of 90° expected for a perfect cube. The atoms defining the three sides of the open cube are almost coplanar (Cu1/O1/Cu5/O10, r.m.s. deviation = 0.0864 Å; Cu5/O7/Cu3/O10, r.m.s. deviation = 0.0588 Å; Cu1/O4/Cu3/O10, r.m.s. deviation = 0.0487 Å) and are irregular with edges of different lengths, *i.e.* for Cu1/O1/Cu5/O10 these are O1—Cu1 = 1.877 (2) Å, O10—Cu1 = 2.004 (2) Å, O1—Cu5 = 2.453 (2) Å and O10—Cu5 = 1.978 (2) Å. Additionally, the dihedral angles values of 78.11 (6), 75.77 (5) and 77.57 (5)° between the sides, two by two, confirm the distortion of the open cube. The bond lengths involving the bridging phenolate oxygen anions and the copper cations are asymmetrical: O1—Cu1 = 1.877 (2) Å and O1—Cu5 = 2. 453 (2) Å; O4—Cu1 = 2.389 (2) Å and O4—Cu3 = 1.896 (2); and O7—Cu5 = 1.889 (2) Å and O7—Cu3 = 2.365 (2) Å. The distances of the μ_3_-bridging O atom to the copper cations are slightly different: O10—Cu1 = 2.005 (2) Å, O10—Cu5 = 1.978 (2) Å and O10—Cu3 = 2.004 (2) Å. The axial bond lengths are longer than the equatorial bond lengths as a result of the Jahn–Teller distortion [Cu1—O4 = 2.389 (2) Å, Cu3—O7 = 2.365 (2) Å and Cu5—O1 = 2.453 (2) Å]. The three copper cations are placed at the vertices of an almost isosceles triangle with distances values of 3.1801 (4) Å (Cu1—Cu5), 3.1823 (4) Å (Cu3—Cu5) and 3.2140 (5) Å (Cu1—Cu3) and angle values of 60.68 (1)° (Cu1—Cu5—Cu3), 59.69 (1)° (Cu5—Cu1—Cu3) and 59.62 (1)° (Cu1—Cu3—Cu5).

For the Cu2 and Cu4 centres, the coordination environments can be best described as slightly distorted square planar with r.m.s. deviations from planarity of 0.0601 Å for Cu2/O2/N2/O3/O11 and 0.0909 Å for Cu4/N4/O5/O1*W*/O6. The τ_4_ (Yang *et al.*, 2007 ▸) values of 0.097 (Cu2) and 0.106 (Cu4) are in accordance with slightly distorted square-planar geometries. For each copper(II) centre (Cu2 and Cu4), the coordination plane and the nearest neighbouring phenyl ring of the ligand are almost co-planar, with respective dihedral angles values of 4.014 (8) and 3.423 (5)°. The copper cation Cu2 is coordinated by one enolato oxygen anion (O2), one phenoxo oxygen anion (O3), one azomethine nitro­gen atom (N2) of the ligand, and one oxygen anion (O11) of an unidentate nitrate group. The Cu2—O2 [1.939 (2) Å], Cu2—O3 [1.855 (2) Å] and Cu2—N2 [1.941 (2) Å] distances are in close proximity to values reported for copper(II) complexes with analogous Schiff base ligands (Popov *et al.*, 2012 ▸; Chen *et al.*, 2004 ▸; Dutta *et al.*, 2020 ▸). The Cu2—O11 bond length [1.9856 (2) Å] is comparable to the distance reported for a nitrato copper complex with square-planar geometry (Thiam *et al.*, 2010 ▸). The *cissoid* angle values are in the range 86.37(9)–94.26 (10)°] and the *transoid* angles are 171.59 (9) and 174.77 (10)°. The Cu4 cation is coordinated by one enolato oxygen anion (O5), one phenoxo oxygen anion (O6), one azomethine nitro­gen atom (N4) of the ligand, and one O atom from a coordinated water mol­ecule. The distances of Cu4 to the coordinated atoms from the ligand [1.916 (2), 1.850 (2) and 1.934 (2) Å] are comparable with those involving Cu2. The Cu4—O1*W* distance value of 1.961 (2) Å is similar to those reported for square-planar copper(II) complexes (Liang *et al.*, 2010 ▸). The *cissoid* angles are in the range 86.56 (8)–95.34 (9)° and the *transoid* angles are 171.10 (9) and 173.69 (9)°. The double-bond character of the C—N bonds [overall values 1.286 (3)–1.295 (3) Å] is indicative of the presence of the imino groups in the three ligands.

## Supra­molecular features   

**Figure 2:**
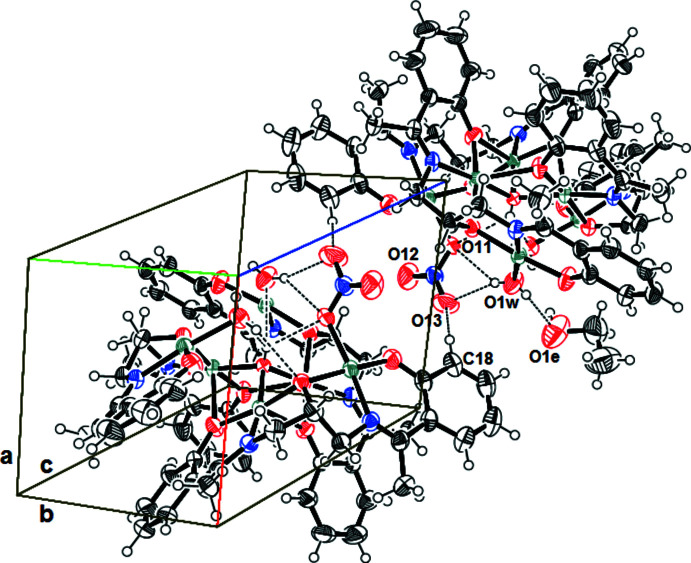
Sheets parallel to the *ac* plane.

**Figure 3:**
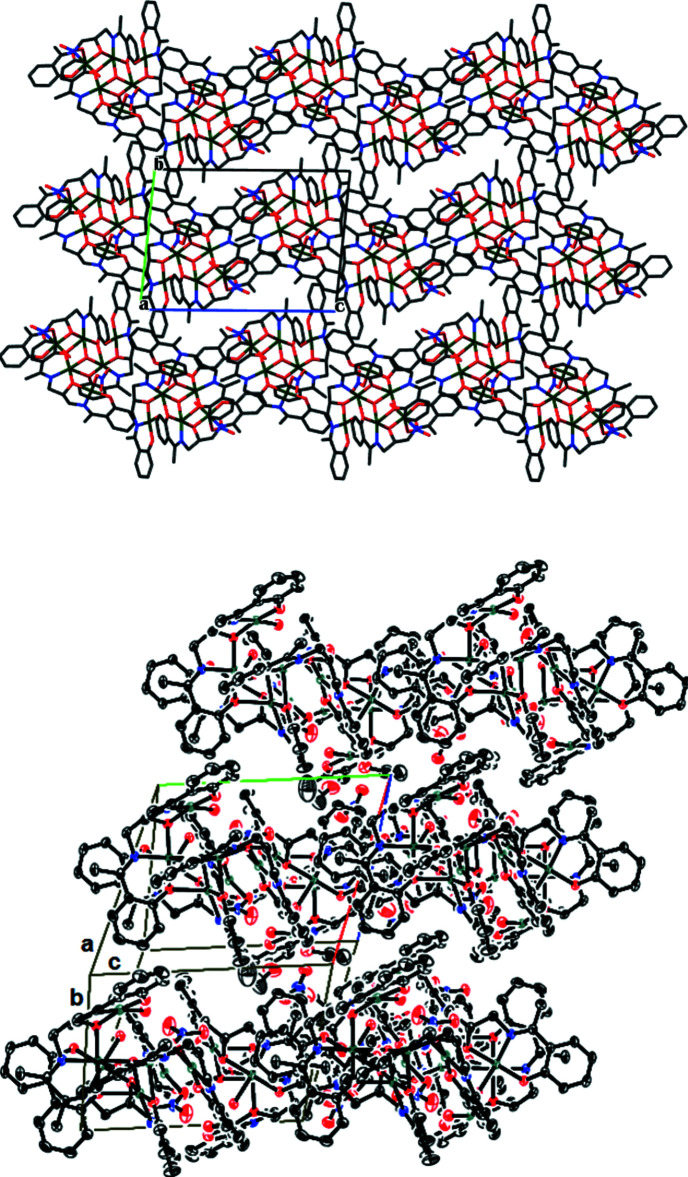
Two views of the zigzagging two-dimensional sheets parallel to the *ac* plane.

In the crystal, intra­molecular and inter­molecular O—H⋯O hydrogen bonds involving the hydroxyl group, the coordinated water mol­ecules and the nitrate and ethanol groups are observed. The complex mol­ecules are inter­connected by inter­molecular hydrogen bonds of type O—H⋯O (O_water_—H⋯O_ethanol_ and O_water_—H⋯O_nitrate_) and C—H⋯O (C_phenolate_—H⋯O_nitrate_) (Fig. 2 ▸, Table 2 ▸). The complex mol­ecules are disposed into zigzagging two-dimensional sheets parallel to the *ac* plane (Fig. 3 ▸). The coordinating water mol­ecules are directed toward the inter­layer region, which is also occupied by the uncoordinated ethanol mol­ecules. Adjacent sheets are linked to one another by hydrogen bonds of type C—H⋯O_ethanol_ or C—H⋯O_nitrate_) (C11—H11*B*⋯O4_ethanol_ and C18—H18⋯O13_nitrate_; Table 3 ▸). The series of inter­molecular and intra­molecular hydrogen bonds stabilize and link the components into a three-dimensional network.

## Database survey   

The ligand *N*,*N*′-bis­[(1-(2-hy­droxy­phen­yl)ethyl­idene)]-2-hy­droxy­propane-1,3-di­amine has been widely used in coordination chemistry. The current release of the CSD (Version 5.42, November 2021 update; Groom *et al.*, 2016 ▸) gave ten hits. Three are complexes of the ligand with Ni^II^ cations [KARPOK and KARPUQ (Liu *et al.*, 2012 ▸); OMOFUS (Banerjee *et al.*, 2011 ▸)]. Three other entries are complexes of Cu^II^ cations [KUKTAM (Basak *et al.*, 2009 ▸), NADDIJ and NADDOP (Osypiuk *et al.*, 2020 ▸)]. In addition, two Co^II^ complexes (OMOFOM and OMOGAZ; Banerjee *et al.*, 2011 ▸), one Fe^II^ (RIDHUJ; Biswas *et al.*, 2013 ▸) and one V^V^ complex (KEWGUQ; Maurya *et al.*, 2013 ▸) have been reported. In all of the ten cases, the ligand acts in a penta­dentate mode through the two soft azomethine nitro­gen atoms, the two hard phenolate oxygen anions and the one hard enolate oxygen anion. In seven cases (KARPOK, KARPUQ, OMOFUS, KUKTAM, NADDIJ, NADDOP and OMOGAZ), the complexes are tetra­nuclear while two dinuclear (OMOFOM and RIDHUJ) and one mononuclear (KEWGUQ) complexes have been reported.

## Synthesis and crystallization   

Reaction of 1-(2-hy­droxy­phen­yl)ethanone and 2-hy­droxy­propane-1,3-di­amine in a 2:1 ratio in ethanol yielded the ligand *N,N* ^’^-bis­{[1-(2-hy­droxy­phen­yl)ethyl­idene]}-2-hy­droxy­propane-1,3-di­amine (H*L*_3_), which was prepared according to a literature method (Song *et al.*, 2003 ▸) with slight modifications. To a solution of 1,3-di­amino­propane-2-ol (0.900 g, 10 mmol) in 25 mL of ethanol was added, dropwise, (2-hy­droxy­phen­yl)ethanone (2.720 g, 20 mmol). The resulting orange mixture was refluxed for 180 min, affording the organic ligand H_3_ *L*. The yellow precipitate that appeared on cooling was recovered by filtration and dried in air. Yield 75%, m.p. 479–480 K. FT–IR (KBr, ν, cm^−1^): 3538 (OH), 3268 (OH), 1605 (C=N), 1538 (C=C), 1528 (C=C), 1455 (C=C), 1247 (C—O), 1043, 760. Analysis calculated for C_19_H_22_N_2_O_3_: C, 69.92; H, 6.79; N, 8.58. Found: C, 69.90; H, 6.76; N, 8.56%. A solution of Cu(NO_3_)_2_·3H_2_O (0.241 g, 1 mmol) in 5 mL of ethanol was added to a solution of H_3_ *L* (0.163 g, 0.5 mmol) in 10 mL of ethanol at room temperature. The initial yellow solution immediately turned dark green and was stirred for 30 min. The mixture was filtered, and the filtrate was kept at 298 K. After one week, light-green crystals suitable for X-ray diffraction were collected and formulated as [Cu_6_ *L*_3_(NO_3_)_2_(OH)(H_2_O)_2_]·3EtOH. FT–IR (KBr, ν, cm^−1^): 1625, 1600, 1540, 1446, 1382, 1304, 1258, 1180, 1120, 1007, 895, 760. Analysis calculated for C_63_H_80_Cu_6_N_8_O_21_: C, 45.40; H, 4.84; N, 6.72. Found: C, 45.38; H, 4.82; N, 6.74%.

## Refinement   

Crystal data, data collection and structure refinement details are summarized in Table 3 ▸. Hydroxyl H atoms were located from difference-Fourier maps and refined. Other H atoms (CH, CH_2_, CH_3_ groups, hydroxyl groups of ethanol mol­ecules and water mol­ecules) were geometrically optimized (C—H = 0.93–0.98 Å, O—H_hy­droxy_ = 0.82 Å and O—H_water_ = 0.86–0.87 Å) and refined as riding with *U*_iso_(H) = 1.2*U*_eq_(C) (1.5 for CH_3_ and OH groups).

## Supplementary Material

Crystal structure: contains datablock(s) I. DOI: 10.1107/S2056989021005570/ex2045sup1.cif

Structure factors: contains datablock(s) I. DOI: 10.1107/S2056989021005570/ex2045Isup2.hkl

CCDC reference: 2086932

Additional supporting information:  crystallographic information; 3D view; checkCIF report

## Figures and Tables

**Table 1:** Selected geometric parameters (Å, °)

Cu3—O10	2.0040 (17)	Cu2—O2	1.9385 (17)
Cu3—O4	1.8963 (17)	Cu2—O3	1.855 (2)
Cu3—O7	2.3648 (17)	Cu2—N2	1.941 (2)
Cu1—O10	2.0043 (19)	Cu6—O14*B*	2.45 (2)
Cu1—O4	2.3893 (17)	Cu6—O8	1.9350 (18)
Cu1—O1	1.8767 (18)	Cu6—O9	1.863 (2)
Cu5—O10	1.9778 (19)	Cu6—O2*W*	2.0273 (19)
Cu5—O1	2.4533 (18)	Cu6—N6	1.942 (2)
Cu4—O5	1.9155 (17)	N1—C7	1.295 (3)
Cu4—O6	1.8496 (19)	N3—C26	1.286 (3)
Cu4—O1*W*	1.961 (2)	N4—C31	1.294 (3)
Cu4—N4	1.934 (2)	N2—C12	1.295 (4)
Cu2—O11	1.986 (2)	N6—C50	1.294 (3)
			
O4—Cu3—O5	170.39 (8)	O3—Cu2—O2	171.60 (8)
N3—Cu3—O10	170.79 (8)	N2—Cu2—O11	174.77 (9)
O1—Cu1—O2	170.60 (8)	Cu3—O10—Cu1	106.62 (9)
N1—Cu1—O10	167.74 (8)	Cu5—O10—Cu3	106.11 (8)
O7—Cu5—O8	171.86 (8)	Cu5—O10—Cu1	105.99 (9)
N5—Cu5—O10	171.32 (9)	Cu3—O4—Cu1	96.50 (7)
O6—Cu4—O5	173.69 (9)	Cu1—O1—Cu5	93.56 (7)
N4—Cu4—O1*W*	171.10 (9)	Cu5—O7—Cu3	96.20 (7)

**Table 2:** Hydrogen-bond geometry (Å, °)

*D*—H⋯*A*	*D*—H	H⋯*A*	*D*⋯*A*	*D*—H⋯*A*
O10—H10⋯O11	0.51 (4)	2.43 (4)	2.854 (3)	142 (6)
O10—H10⋯O2*W*	0.51 (4)	2.75 (3)	2.910 (3)	103 (4)
O10—H10⋯O1*W*	0.51 (4)	2.73 (4)	3.155 (3)	143 (6)
O1*W*—H1*WA*⋯O1*E*	0.86	2.26	2.625 (4)	106
O1*W*—H1*WB*⋯O13	0.86	2.21	2.876 (4)	135
C11—H11*B*⋯O4*E* ^i^	0.97	2.31	3.280 (4)	174
C18—H18⋯O13^ii^	0.93	2.54	3.328 (4)	143
O4*E*—H4*E*⋯N202	0.82	2.66	3.447 (5)	161
O4*E*—H4*E*⋯O16*B*	0.82	2.45	3.13 (2)	141
O4*E*—H4*E*⋯O15*B*	0.82	2.12	2.87 (3)	151

Symmetry codes: (i) -x+1, -y+1, -z+1; (ii) -x+1, -y+1, -z.

**Table 3:** Experimental details

Crystal data	
Chemical formula	[Cu_6_(C_19_H_19_N_2_O_3_)_3_(NO_3_)_2_(OH)(H_2_O)_2_]·3C_2_H_6_O
*M*_r_	1666.59
Crystal system, space group	Triclinic, *P*\overline{1}
Temperature (K)	293
*a*, *b*, *c* (Å)	13.6406 (5), 14.0568 (5), 18.5907 (7)
α, β, γ (°)	83.626 (3), 86.186 (3), 72.288 (3)
*V* (Å^3^)	3372.7 (2)
*Z*	2
Radiation type	Mo *K*α
μ (mm^−1^)	1.94
Crystal size (mm)	0.3 × 0.2 × 0.1

Data collection	
Diffractometer	Nonius KappaCCD
Absorption correction	Multi-scan (*SADABS*; Sheldrick, 1996 ▸)
*T*_min_, *T*_max_	0.967, 1.000
No. of measured, independent and observed [*I* > 2σ(*I*)] reflections	73743, 14284, 12395
*R*_int_	0.033
(sin θ/λ)_max_ (Å^−1^)	0.633

Refinement	
*R*[*F*^2^ > 2σ(*F* ^2^)], *wR*(*F* ^2^), *S*	0.033, 0.091, 1.04
No. of reflections	14284
No. of parameters	929
No. of restraints	3
H-atom treatment	H atoms treated by a mixture of independent and constrained refinement
Δρ_max_, Δρ_min_ (e Å^−3^)	0.82, −0.56

Computer programs: *APEX3* and *SAINT* (Bruker, 2016 ▸), *SHELXT2018/2* (Sheldrick, 2015*a* ▸), *SHELXL* (Sheldrick, 2015*b* ▸) and *OLEX2* (Dolomanov *et al.*, 2009 ▸).

## supplementary crystallographic information

### Crystal data

**Table:**

[Cu_6_(C_19_H_19_N_2_O_3_)_3_(NO_3_)_2_(OH)(H_2_O)_2_]·3C_2_H_6_O	*Z* = 2
*M**_r_* = 1666.59	*F*(000) = 1712
Triclinic, *P*1	*D*_x_ = 1.641 Mg m^−^^3^
*a* = 13.6406 (5) Å	Mo *K*α radiation, λ = 0.71073 Å
*b* = 14.0568 (5) Å	Cell parameters from 5100 reflections
*c* = 18.5907 (7) Å	θ = 2.4–28.6°
α = 83.626 (3)°	µ = 1.94 mm^−^^1^
β = 86.186 (3)°	*T* = 293 K
γ = 72.288 (3)°	Prismatic, green
*V* = 3372.7 (2) Å^3^	0.3 × 0.2 × 0.1 mm

### Data collection

**Table:**

KappaCCD diffractometer	12395 reflections with *I* > 2σ(*I*)
Detector resolution: 9 pixels mm^-1^	*R*_int_ = 0.033
CCD scans	θ_max_ = 26.7°, θ_min_ = 2.8°
Absorption correction: multi-scan	*h* = −17→17
*T*_min_ = 0.967, *T*_max_ = 1.000	*k* = −17→17
73743 measured reflections	*l* = −23→23
14284 independent reflections	

### Refinement

**Table:**

Refinement on *F*^2^	3 restraints
Least-squares matrix: full	Hydrogen site location: mixed
*R*[*F*^2^ > 2σ(*F*^2^)] = 0.033	H atoms treated by a mixture of independent and constrained refinement
*wR*(*F*^2^) = 0.091	*w* = 1/[σ^2^(*F*_o_^2^) + (0.0432*P*)^2^ + 3.2696*P*] where *P* = (*F*_o_^2^ + 2*F*_c_^2^)/3
*S* = 1.04	(Δ/σ)_max_ = 0.001
14284 reflections	Δρ_max_ = 0.82 e Å^−^^3^
929 parameters	Δρ_min_ = −0.56 e Å^−^^3^

### Special details

**Table:**

Geometry. All esds (except the esd in the dihedral angle between two l.s. planes) are estimated using the full covariance matrix. The cell esds are taken into account individually in the estimation of esds in distances, angles and torsion angles; correlations between esds in cell parameters are only used when they are defined by crystal symmetry. An approximate (isotropic) treatment of cell esds is used for estimating esds involving l.s. planes.

### Fractional atomic coordinates and isotropic or equivalent isotropic displacement parameters (Å^2^)

**Table:**

	*x*	*y*	*z*	*U*_iso_*/*U*_eq_	Occ. (<1)
Cu3	0.26915 (2)	0.40125 (2)	0.15888 (2)	0.03091 (7)	
Cu1	0.27776 (2)	0.42624 (2)	0.32790 (2)	0.03074 (7)	
Cu5	0.34276 (2)	0.20896 (2)	0.27233 (2)	0.03197 (7)	
Cu4	0.45315 (2)	0.20182 (2)	0.10809 (2)	0.03414 (8)	
Cu2	0.34537 (2)	0.58294 (2)	0.20408 (2)	0.03620 (8)	
O11	0.47163 (16)	0.47175 (15)	0.18459 (13)	0.0555 (6)	
N102	0.54911 (19)	0.49292 (17)	0.15507 (13)	0.0430 (5)	
O12	0.5566 (2)	0.57631 (18)	0.15453 (17)	0.0771 (8)	
O13	0.6141 (2)	0.4241 (2)	0.12800 (19)	0.0958 (11)	
Cu6	0.46902 (2)	0.25221 (2)	0.40915 (2)	0.03727 (8)	
O14B	0.6475 (14)	0.152 (2)	0.4307 (18)	0.074 (6)	0.43 (8)
N202	0.6961 (3)	0.1605 (3)	0.4849 (2)	0.0743 (9)	
O16B	0.7637 (19)	0.203 (3)	0.4682 (15)	0.087 (7)	0.43 (8)
O15B	0.708 (5)	0.102 (5)	0.5418 (17)	0.143 (11)	0.43 (8)
O10	0.35231 (15)	0.34640 (13)	0.24727 (10)	0.0291 (4)	
H10	0.384 (2)	0.350 (4)	0.232 (2)	0.073 (17)*	
O2	0.34884 (13)	0.52783 (13)	0.30453 (9)	0.0348 (4)	
O5	0.37695 (14)	0.34071 (12)	0.09029 (9)	0.0347 (4)	
O8	0.45828 (14)	0.16793 (13)	0.33636 (9)	0.0364 (4)	
O4	0.17301 (13)	0.47876 (13)	0.22424 (9)	0.0374 (4)	
O1	0.22912 (15)	0.31642 (14)	0.35827 (10)	0.0415 (4)	
O7	0.24464 (15)	0.24428 (13)	0.19929 (10)	0.0397 (4)	
O9	0.46067 (17)	0.34675 (16)	0.47384 (10)	0.0495 (5)	
O3	0.33169 (16)	0.62254 (15)	0.10579 (11)	0.0482 (5)	
O2W	0.52462 (16)	0.34018 (16)	0.33383 (11)	0.0478 (5)	
H2WA	0.483126	0.400782	0.330414	0.072*	
H2WB	0.524712	0.321109	0.291044	0.072*	
O6	0.52042 (16)	0.06843 (14)	0.13553 (12)	0.0483 (5)	
O1W	0.55379 (17)	0.24409 (15)	0.15701 (14)	0.0562 (6)	
H1WA	0.552628	0.224827	0.202374	0.084*	
H1WB	0.535206	0.308242	0.155417	0.084*	
N5	0.34465 (17)	0.06909 (15)	0.28312 (12)	0.0366 (5)	
N1	0.23171 (16)	0.48927 (16)	0.41787 (11)	0.0354 (4)	
N3	0.19297 (17)	0.47496 (15)	0.07387 (11)	0.0353 (4)	
N4	0.35642 (17)	0.17511 (15)	0.04761 (11)	0.0356 (4)	
N2	0.22263 (17)	0.68655 (16)	0.23230 (12)	0.0376 (5)	
N6	0.40344 (17)	0.17147 (17)	0.47557 (12)	0.0387 (5)	
C7	0.18607 (19)	0.4560 (2)	0.47399 (13)	0.0371 (5)	
C20	0.07635 (19)	0.52770 (19)	0.21000 (14)	0.0352 (5)	
C1	0.1607 (2)	0.31345 (19)	0.41129 (14)	0.0380 (5)	
C26	0.1061 (2)	0.54451 (18)	0.07321 (13)	0.0363 (5)	
C10	0.2991 (2)	0.6107 (2)	0.34627 (14)	0.0395 (6)	
H10A	0.344598	0.652585	0.347556	0.047*	
C25	0.0398 (2)	0.5632 (2)	0.13906 (15)	0.0391 (6)	
C48	0.4605 (2)	0.07339 (19)	0.37428 (15)	0.0403 (6)	
H48	0.530044	0.040509	0.391873	0.048*	
C11	0.2003 (2)	0.6723 (2)	0.31042 (14)	0.0413 (6)	
H11A	0.148399	0.637664	0.319344	0.050*	
H11B	0.174402	0.736740	0.330030	0.050*	
C45	0.2881 (2)	0.0286 (2)	0.25189 (14)	0.0402 (6)	
C39	0.1788 (2)	0.1953 (2)	0.19148 (13)	0.0387 (6)	
C12	0.1539 (2)	0.74659 (19)	0.19013 (15)	0.0406 (6)	
C33	0.4114 (2)	−0.00413 (19)	0.07486 (14)	0.0369 (5)	
C38	0.4955 (2)	−0.00899 (19)	0.11758 (14)	0.0393 (6)	
C14	0.1755 (2)	0.7628 (2)	0.11249 (15)	0.0432 (6)	
C31	0.3412 (2)	0.0897 (2)	0.04257 (14)	0.0388 (6)	
C6	0.1395 (2)	0.3760 (2)	0.46934 (14)	0.0389 (6)	
C29	0.3363 (2)	0.35247 (19)	0.02049 (13)	0.0385 (6)	
H29	0.392788	0.346029	−0.015882	0.046*	
C52	0.3861 (2)	0.2683 (2)	0.57706 (14)	0.0425 (6)	
C57	0.4333 (2)	0.3393 (2)	0.54279 (15)	0.0430 (6)	
C50	0.3630 (2)	0.1919 (2)	0.53911 (14)	0.0407 (6)	
C30	0.2873 (2)	0.26973 (19)	0.01504 (14)	0.0403 (6)	
H30A	0.220724	0.285068	0.040503	0.048*	
H30B	0.277218	0.264110	−0.035304	0.048*	
C28	0.2603 (2)	0.45634 (19)	0.00831 (14)	0.0430 (6)	
H28A	0.296647	0.506237	−0.000681	0.052*	
H28B	0.219496	0.460380	−0.033360	0.052*	
C49	0.3854 (2)	0.0910 (2)	0.43897 (15)	0.0420 (6)	
H49A	0.315113	0.110708	0.423144	0.050*	
H49B	0.396950	0.030161	0.471690	0.050*	
C47	0.4357 (2)	0.0077 (2)	0.32343 (16)	0.0447 (6)	
H47A	0.493601	−0.016513	0.290148	0.054*	
H47B	0.421140	−0.049776	0.350687	0.054*	
C34	0.3961 (2)	−0.0959 (2)	0.06230 (17)	0.0456 (6)	
H34	0.341428	−0.094117	0.034323	0.055*	
C9	0.2783 (2)	0.5696 (2)	0.42281 (15)	0.0433 (6)	
H9A	0.342047	0.543434	0.448457	0.052*	
H9B	0.231806	0.622368	0.448939	0.052*	
C44	0.1967 (2)	0.0912 (2)	0.21363 (15)	0.0433 (6)	
C19	0.2634 (2)	0.7014 (2)	0.07612 (15)	0.0430 (6)	
C37	0.5581 (2)	−0.1043 (2)	0.14436 (16)	0.0465 (6)	
H37	0.613835	−0.108570	0.172097	0.056*	
C40	0.0862 (2)	0.2485 (2)	0.15771 (16)	0.0507 (7)	
H40	0.074005	0.316381	0.142863	0.061*	
C21	0.0055 (2)	0.5483 (2)	0.26906 (16)	0.0500 (7)	
H21	0.027877	0.524611	0.315701	0.060*	
C27	0.0697 (3)	0.6096 (2)	0.00429 (16)	0.0498 (7)	
H27A	0.027849	0.674821	0.015851	0.075*	
H27B	0.029929	0.579511	−0.021464	0.075*	
H27C	0.128140	0.615913	−0.025405	0.075*	
C35	0.4583 (2)	−0.1876 (2)	0.08949 (18)	0.0503 (7)	
H35	0.445766	−0.246387	0.080161	0.060*	
C56	0.4501 (3)	0.4105 (3)	0.58486 (17)	0.0558 (8)	
H56	0.478737	0.458896	0.562606	0.067*	
C5	0.0698 (2)	0.3587 (2)	0.52516 (16)	0.0508 (7)	
H5	0.056342	0.397695	0.564032	0.061*	
C8	0.1792 (3)	0.4997 (2)	0.54539 (15)	0.0510 (7)	
H8A	0.197019	0.446278	0.583647	0.076*	
H8B	0.110182	0.541525	0.553991	0.076*	
H8C	0.225958	0.538942	0.543959	0.076*	
C36	0.5394 (3)	−0.1909 (2)	0.13075 (17)	0.0514 (7)	
H36	0.582000	−0.252565	0.149631	0.062*	
C53	0.3612 (3)	0.2718 (3)	0.65203 (17)	0.0572 (8)	
H53	0.330892	0.225414	0.675595	0.069*	
C2	0.1082 (3)	0.2418 (2)	0.41137 (19)	0.0539 (7)	
H2	0.119918	0.201860	0.373136	0.065*	
C18	0.2780 (3)	0.7253 (3)	0.00078 (16)	0.0537 (7)	
H18	0.334628	0.685543	−0.023813	0.064*	
C4	0.0214 (3)	0.2868 (3)	0.5242 (2)	0.0618 (9)	
H4	−0.023576	0.276928	0.562031	0.074*	
C51	0.2902 (2)	0.1381 (3)	0.57490 (18)	0.0547 (8)	
H51A	0.230334	0.185992	0.593733	0.082*	
H51B	0.323795	0.091260	0.613790	0.082*	
H51C	0.269921	0.102823	0.540051	0.082*	
C13	0.0494 (2)	0.8001 (2)	0.22090 (19)	0.0543 (7)	
H13A	0.000138	0.817472	0.183404	0.081*	
H13B	0.051906	0.859959	0.240006	0.081*	
H13C	0.029410	0.756948	0.258935	0.081*	
C17	0.2110 (3)	0.8051 (3)	−0.03647 (18)	0.0633 (9)	
H17	0.222439	0.819080	−0.085837	0.076*	
C46	0.3131 (3)	−0.0840 (2)	0.25508 (18)	0.0549 (8)	
H46A	0.288793	−0.101614	0.212633	0.082*	
H46B	0.280145	−0.107984	0.297433	0.082*	
H46C	0.386285	−0.114111	0.257190	0.082*	
C55	0.4253 (3)	0.4100 (3)	0.65739 (18)	0.0658 (10)	
H55	0.439053	0.456522	0.683947	0.079*	
C24	−0.0643 (2)	0.6184 (3)	0.13261 (19)	0.0600 (9)	
H24	−0.089030	0.642032	0.086524	0.072*	
C3	0.0399 (3)	0.2292 (3)	0.4666 (2)	0.0630 (9)	
H3	0.005786	0.181409	0.465104	0.076*	
C43	0.1199 (3)	0.0485 (3)	0.1994 (2)	0.0666 (10)	
H43	0.130776	−0.019621	0.212685	0.080*	
C15	0.1093 (3)	0.8438 (2)	0.07133 (19)	0.0606 (8)	
H15	0.051529	0.884516	0.094237	0.073*	
C22	−0.0961 (3)	0.6029 (3)	0.2594 (2)	0.0684 (10)	
H22	−0.141280	0.615301	0.299338	0.082*	
C16	0.1263 (3)	0.8651 (3)	−0.0012 (2)	0.0701 (10)	
H16	0.081091	0.919827	−0.026654	0.084*	
C32	0.2496 (3)	0.0849 (3)	0.0041 (2)	0.0599 (8)	
H32A	0.214545	0.044365	0.034003	0.090*	
H32B	0.272193	0.055764	−0.040759	0.090*	
H32C	0.203461	0.151345	−0.005629	0.090*	
C41	0.0132 (3)	0.2032 (3)	0.1459 (2)	0.0661 (10)	
H41	−0.047657	0.240543	0.123863	0.079*	
C54	0.3801 (3)	0.3410 (3)	0.69121 (18)	0.0684 (10)	
H54	0.362483	0.341361	0.740396	0.082*	
C42	0.0301 (3)	0.1023 (3)	0.1669 (2)	0.0790 (13)	
H42	−0.018959	0.071318	0.159012	0.095*	
C23	−0.1314 (3)	0.6392 (4)	0.1910 (2)	0.0774 (12)	
H23	−0.199653	0.677280	0.184504	0.093*	
O1E	0.7184 (3)	0.1473 (3)	0.2304 (2)	0.1102 (12)	
H1E	0.696426	0.187818	0.260536	0.165*	
C2E	0.7195 (4)	0.0498 (4)	0.2618 (3)	0.0964 (15)	
H2EA	0.649987	0.044852	0.267654	0.116*	
H2EB	0.750187	0.035764	0.308862	0.116*	
C3E	0.7804 (5)	−0.0208 (5)	0.2123 (4)	0.133 (2)	
H3EA	0.780131	−0.087531	0.229966	0.200*	
H3EB	0.849951	−0.017689	0.208977	0.200*	
H3EC	0.751412	−0.003753	0.165191	0.200*	
O4E	0.8827 (3)	0.1187 (3)	0.6110 (2)	0.1057 (12)	
H4E	0.829086	0.136427	0.588792	0.159*	
C5E	0.9362 (5)	0.0241 (4)	0.5980 (3)	0.1034 (17)	
H5EA	0.937397	0.017419	0.546563	0.124*	
H5EB	0.902534	−0.022216	0.623292	0.124*	
C6E	1.0416 (6)	−0.0011 (5)	0.6222 (4)	0.137 (3)	
H6EA	1.080592	−0.065847	0.607859	0.206*	
H6EB	1.040758	−0.002482	0.673943	0.206*	
H6EC	1.072798	0.048381	0.600560	0.206*	
O7E	0.7319 (3)	0.2834 (4)	0.3279 (2)	0.1182 (13)	
H7E	0.695981	0.316198	0.359088	0.177*	
C8E	0.8232 (9)	0.3177 (8)	0.3102 (4)	0.172 (4)	
H8EA	0.836358	0.352431	0.349068	0.207*	
H8EB	0.883662	0.261511	0.301851	0.207*	
C9E	0.7977 (8)	0.3837 (7)	0.2462 (6)	0.229 (6)	
H9EA	0.778579	0.349547	0.209975	0.343*	
H9EB	0.856115	0.405052	0.228575	0.343*	
H9EC	0.741038	0.441138	0.256569	0.343*	
O16A	0.750 (2)	0.216 (2)	0.4779 (17)	0.112 (7)	0.57 (8)
O15A	0.675 (2)	0.138 (2)	0.5466 (7)	0.115 (5)	0.57 (8)
O14A	0.6499 (14)	0.1441 (17)	0.4376 (16)	0.085 (6)	0.57 (8)

### Atomic displacement parameters (Å^2^)

**Table:**

	*U*^11^	*U*^22^	*U*^33^	*U*^12^	*U*^13^	*U*^23^
Cu3	0.03783 (16)	0.02639 (14)	0.02544 (14)	−0.00612 (12)	−0.00082 (11)	0.00037 (11)
Cu1	0.03459 (15)	0.02884 (15)	0.02828 (14)	−0.00970 (12)	0.00320 (11)	−0.00249 (11)
Cu5	0.03869 (16)	0.02569 (14)	0.03087 (15)	−0.01019 (12)	−0.00316 (12)	0.00283 (11)
Cu4	0.03985 (16)	0.02640 (14)	0.03622 (16)	−0.01025 (12)	−0.00088 (12)	−0.00257 (12)
Cu2	0.03940 (17)	0.02984 (15)	0.03844 (16)	−0.01114 (13)	0.00403 (13)	−0.00033 (12)
O11	0.0483 (12)	0.0390 (11)	0.0702 (14)	−0.0103 (9)	0.0211 (10)	0.0112 (10)
N102	0.0496 (13)	0.0350 (12)	0.0455 (13)	−0.0153 (10)	0.0081 (10)	−0.0062 (10)
O12	0.0838 (18)	0.0415 (13)	0.115 (2)	−0.0322 (13)	−0.0159 (16)	−0.0004 (13)
O13	0.085 (2)	0.0660 (17)	0.124 (3)	−0.0127 (15)	0.0622 (19)	−0.0254 (17)
Cu6	0.03732 (16)	0.04047 (17)	0.03351 (16)	−0.01265 (13)	−0.00105 (12)	0.00147 (13)
O14B	0.036 (8)	0.096 (13)	0.082 (9)	0.000 (7)	−0.007 (6)	−0.025 (8)
N202	0.062 (2)	0.079 (2)	0.083 (3)	−0.0231 (18)	−0.0093 (18)	−0.002 (2)
O16B	0.064 (8)	0.143 (18)	0.073 (8)	−0.060 (8)	−0.008 (5)	−0.004 (9)
O15B	0.15 (2)	0.15 (2)	0.148 (16)	−0.09 (2)	−0.043 (12)	0.053 (12)
O10	0.0297 (8)	0.0275 (8)	0.0283 (8)	−0.0077 (7)	0.0015 (7)	0.0012 (6)
O2	0.0359 (9)	0.0329 (9)	0.0366 (9)	−0.0124 (7)	0.0012 (7)	−0.0030 (7)
O5	0.0433 (10)	0.0277 (8)	0.0313 (8)	−0.0088 (7)	0.0034 (7)	−0.0034 (7)
O8	0.0381 (9)	0.0327 (9)	0.0360 (9)	−0.0093 (7)	−0.0032 (7)	0.0037 (7)
O4	0.0376 (9)	0.0372 (9)	0.0304 (8)	−0.0004 (7)	−0.0027 (7)	−0.0036 (7)
O1	0.0538 (11)	0.0369 (10)	0.0358 (9)	−0.0192 (8)	0.0127 (8)	−0.0045 (7)
O7	0.0506 (11)	0.0328 (9)	0.0392 (9)	−0.0189 (8)	−0.0118 (8)	0.0062 (7)
O9	0.0616 (13)	0.0546 (12)	0.0377 (10)	−0.0262 (10)	0.0034 (9)	−0.0053 (9)
O3	0.0530 (12)	0.0456 (11)	0.0401 (10)	−0.0093 (9)	0.0065 (9)	0.0002 (8)
O2W	0.0545 (12)	0.0488 (11)	0.0429 (10)	−0.0219 (10)	0.0029 (9)	−0.0001 (9)
O6	0.0573 (12)	0.0292 (9)	0.0592 (12)	−0.0113 (9)	−0.0216 (10)	−0.0006 (8)
O1W	0.0556 (12)	0.0344 (10)	0.0829 (16)	−0.0175 (9)	−0.0196 (11)	−0.0020 (10)
N5	0.0442 (12)	0.0274 (10)	0.0369 (11)	−0.0106 (9)	−0.0014 (9)	0.0014 (8)
N1	0.0379 (11)	0.0364 (11)	0.0304 (10)	−0.0085 (9)	−0.0008 (8)	−0.0047 (8)
N3	0.0476 (12)	0.0284 (10)	0.0276 (10)	−0.0096 (9)	−0.0022 (9)	0.0019 (8)
N4	0.0445 (12)	0.0296 (10)	0.0318 (10)	−0.0102 (9)	−0.0018 (9)	−0.0019 (8)
N2	0.0427 (12)	0.0299 (10)	0.0402 (11)	−0.0108 (9)	−0.0006 (9)	−0.0033 (9)
N6	0.0386 (11)	0.0389 (12)	0.0352 (11)	−0.0091 (9)	−0.0035 (9)	0.0047 (9)
C7	0.0342 (12)	0.0395 (13)	0.0304 (12)	−0.0007 (10)	−0.0018 (10)	−0.0013 (10)
C20	0.0348 (12)	0.0322 (12)	0.0376 (13)	−0.0087 (10)	−0.0035 (10)	−0.0012 (10)
C1	0.0375 (13)	0.0342 (13)	0.0379 (13)	−0.0081 (10)	0.0022 (10)	0.0060 (10)
C26	0.0467 (14)	0.0296 (12)	0.0339 (12)	−0.0131 (11)	−0.0094 (11)	0.0014 (10)
C10	0.0474 (15)	0.0369 (13)	0.0389 (13)	−0.0180 (12)	0.0003 (11)	−0.0094 (11)
C25	0.0388 (13)	0.0359 (13)	0.0408 (14)	−0.0101 (11)	−0.0055 (11)	0.0023 (11)
C48	0.0390 (13)	0.0315 (13)	0.0437 (14)	−0.0030 (10)	−0.0060 (11)	0.0058 (11)
C11	0.0505 (15)	0.0340 (13)	0.0386 (13)	−0.0113 (11)	0.0049 (11)	−0.0076 (11)
C45	0.0535 (16)	0.0326 (13)	0.0358 (13)	−0.0179 (12)	0.0075 (11)	−0.0003 (10)
C39	0.0503 (15)	0.0436 (14)	0.0280 (12)	−0.0239 (12)	−0.0012 (10)	0.0005 (10)
C12	0.0466 (15)	0.0285 (12)	0.0482 (15)	−0.0128 (11)	−0.0018 (12)	−0.0053 (11)
C33	0.0439 (14)	0.0304 (12)	0.0377 (13)	−0.0138 (11)	0.0037 (11)	−0.0043 (10)
C38	0.0481 (15)	0.0288 (12)	0.0402 (13)	−0.0118 (11)	0.0009 (11)	−0.0015 (10)
C14	0.0551 (16)	0.0323 (13)	0.0439 (14)	−0.0155 (12)	−0.0076 (12)	−0.0002 (11)
C31	0.0459 (14)	0.0348 (13)	0.0374 (13)	−0.0143 (11)	0.0007 (11)	−0.0053 (10)
C6	0.0340 (13)	0.0401 (14)	0.0348 (13)	−0.0031 (11)	0.0024 (10)	0.0047 (10)
C29	0.0533 (15)	0.0317 (12)	0.0281 (12)	−0.0112 (11)	0.0060 (11)	−0.0008 (10)
C52	0.0358 (13)	0.0501 (16)	0.0349 (13)	−0.0042 (12)	−0.0035 (10)	0.0006 (11)
C57	0.0344 (13)	0.0557 (17)	0.0366 (13)	−0.0095 (12)	−0.0043 (11)	−0.0042 (12)
C50	0.0340 (13)	0.0411 (14)	0.0383 (13)	−0.0028 (11)	−0.0027 (10)	0.0092 (11)
C30	0.0522 (15)	0.0330 (13)	0.0322 (12)	−0.0069 (11)	−0.0061 (11)	−0.0019 (10)
C28	0.0644 (18)	0.0317 (13)	0.0279 (12)	−0.0103 (12)	0.0027 (12)	0.0038 (10)
C49	0.0467 (15)	0.0374 (14)	0.0398 (14)	−0.0125 (12)	−0.0030 (11)	0.0065 (11)
C47	0.0490 (16)	0.0299 (13)	0.0477 (15)	−0.0025 (11)	−0.0013 (12)	0.0015 (11)
C34	0.0503 (16)	0.0335 (14)	0.0556 (17)	−0.0158 (12)	0.0020 (13)	−0.0082 (12)
C9	0.0516 (16)	0.0431 (15)	0.0382 (14)	−0.0161 (12)	−0.0047 (12)	−0.0096 (11)
C44	0.0564 (17)	0.0439 (15)	0.0365 (13)	−0.0267 (13)	−0.0019 (12)	0.0008 (11)
C19	0.0552 (16)	0.0393 (14)	0.0400 (14)	−0.0231 (13)	−0.0020 (12)	−0.0013 (11)
C37	0.0517 (16)	0.0336 (14)	0.0506 (16)	−0.0079 (12)	−0.0053 (13)	−0.0005 (12)
C40	0.0572 (18)	0.0539 (17)	0.0438 (15)	−0.0236 (15)	−0.0126 (13)	0.0093 (13)
C21	0.0418 (15)	0.0606 (19)	0.0417 (15)	−0.0098 (13)	0.0017 (12)	0.0031 (13)
C27	0.0617 (18)	0.0384 (15)	0.0429 (15)	−0.0074 (13)	−0.0128 (13)	0.0086 (12)
C35	0.0557 (17)	0.0277 (13)	0.069 (2)	−0.0148 (12)	0.0074 (15)	−0.0098 (13)
C56	0.0505 (17)	0.074 (2)	0.0495 (17)	−0.0266 (16)	−0.0019 (14)	−0.0121 (15)
C5	0.0443 (16)	0.0526 (17)	0.0445 (15)	−0.0036 (13)	0.0123 (12)	0.0031 (13)
C8	0.0582 (18)	0.0596 (18)	0.0302 (13)	−0.0099 (15)	0.0014 (12)	−0.0072 (12)
C36	0.0610 (19)	0.0292 (13)	0.0584 (18)	−0.0076 (13)	0.0040 (15)	−0.0004 (12)
C53	0.0567 (19)	0.069 (2)	0.0405 (16)	−0.0149 (16)	0.0021 (13)	0.0031 (15)
C2	0.0582 (18)	0.0448 (16)	0.0604 (19)	−0.0217 (14)	0.0079 (15)	−0.0001 (14)
C18	0.073 (2)	0.0538 (18)	0.0413 (15)	−0.0307 (16)	0.0010 (14)	−0.0009 (13)
C4	0.0477 (17)	0.0582 (19)	0.069 (2)	−0.0117 (15)	0.0221 (16)	0.0105 (17)
C51	0.0522 (17)	0.0555 (18)	0.0509 (17)	−0.0146 (14)	0.0097 (14)	0.0066 (14)
C13	0.0498 (17)	0.0438 (16)	0.0611 (19)	−0.0030 (13)	0.0000 (14)	−0.0024 (14)
C17	0.097 (3)	0.059 (2)	0.0423 (16)	−0.037 (2)	−0.0127 (17)	0.0060 (15)
C46	0.074 (2)	0.0353 (15)	0.0597 (19)	−0.0242 (15)	0.0043 (16)	−0.0052 (13)
C55	0.064 (2)	0.096 (3)	0.0469 (18)	−0.033 (2)	−0.0036 (15)	−0.0220 (18)
C24	0.0399 (16)	0.077 (2)	0.0536 (18)	−0.0075 (15)	−0.0102 (14)	0.0090 (16)
C3	0.0552 (19)	0.0545 (19)	0.082 (2)	−0.0274 (16)	0.0138 (17)	0.0050 (17)
C43	0.083 (2)	0.061 (2)	0.071 (2)	−0.048 (2)	−0.0197 (19)	0.0135 (17)
C15	0.075 (2)	0.0427 (17)	0.0579 (19)	−0.0069 (15)	−0.0136 (17)	−0.0016 (14)
C22	0.0390 (16)	0.094 (3)	0.059 (2)	−0.0081 (17)	0.0104 (14)	0.0062 (19)
C16	0.098 (3)	0.0488 (19)	0.058 (2)	−0.0136 (19)	−0.026 (2)	0.0095 (16)
C32	0.063 (2)	0.0502 (18)	0.072 (2)	−0.0216 (16)	−0.0243 (17)	−0.0025 (16)
C41	0.064 (2)	0.080 (2)	0.063 (2)	−0.0385 (19)	−0.0237 (17)	0.0187 (18)
C54	0.075 (2)	0.098 (3)	0.0357 (16)	−0.030 (2)	0.0003 (15)	−0.0097 (17)
C42	0.086 (3)	0.090 (3)	0.083 (3)	−0.063 (2)	−0.035 (2)	0.023 (2)
C23	0.0350 (16)	0.104 (3)	0.075 (2)	−0.0010 (18)	−0.0054 (16)	0.012 (2)
O1E	0.116 (3)	0.078 (2)	0.135 (3)	−0.0183 (19)	−0.056 (2)	−0.004 (2)
C2E	0.104 (4)	0.096 (4)	0.085 (3)	−0.030 (3)	−0.010 (3)	0.017 (3)
C3E	0.089 (4)	0.109 (5)	0.207 (8)	−0.025 (3)	0.013 (4)	−0.054 (5)
O4E	0.103 (3)	0.088 (2)	0.129 (3)	−0.0157 (19)	−0.024 (2)	−0.052 (2)
C5E	0.136 (5)	0.077 (3)	0.095 (4)	−0.026 (3)	0.011 (3)	−0.026 (3)
C6E	0.137 (6)	0.127 (5)	0.118 (5)	0.012 (4)	−0.026 (4)	−0.020 (4)
O7E	0.087 (2)	0.172 (4)	0.103 (3)	−0.061 (3)	−0.001 (2)	0.016 (3)
C8E	0.264 (12)	0.177 (9)	0.114 (6)	−0.123 (9)	0.026 (7)	−0.033 (5)
C9E	0.174 (10)	0.172 (10)	0.340 (18)	−0.053 (8)	0.032 (11)	−0.045 (11)
O16A	0.134 (14)	0.103 (8)	0.126 (13)	−0.070 (8)	−0.008 (9)	−0.025 (8)
O15A	0.131 (10)	0.146 (12)	0.061 (8)	−0.043 (10)	0.000 (5)	0.026 (5)
O14A	0.079 (10)	0.071 (7)	0.111 (12)	−0.027 (6)	−0.028 (7)	−0.015 (6)

### Geometric parameters (Å, º)

**Table:**

Cu3—O10	2.0040 (17)	C7—C8	1.509 (4)
Cu3—O5	1.9346 (17)	C20—C25	1.424 (4)
Cu3—O4	1.8963 (17)	C20—C21	1.408 (4)
Cu3—O7	2.3648 (17)	C1—C6	1.427 (4)
Cu3—N3	1.962 (2)	C1—C2	1.402 (4)
Cu1—O10	2.0043 (19)	C26—C25	1.471 (4)
Cu1—O2	1.9538 (17)	C26—C27	1.508 (3)
Cu1—O4	2.3893 (17)	C10—C11	1.514 (4)
Cu1—O1	1.8767 (18)	C10—C9	1.518 (4)
Cu1—N1	1.956 (2)	C25—C24	1.402 (4)
Cu5—O10	1.9778 (19)	C48—C49	1.518 (4)
Cu5—O8	1.9434 (18)	C48—C47	1.513 (4)
Cu5—O1	2.4533 (18)	C45—C44	1.464 (4)
Cu5—O7	1.8894 (18)	C45—C46	1.510 (4)
Cu5—N5	1.946 (2)	C39—C44	1.426 (4)
Cu4—O5	1.9155 (17)	C39—C40	1.405 (4)
Cu4—O6	1.8496 (19)	C12—C14	1.461 (4)
Cu4—O1W	1.961 (2)	C12—C13	1.503 (4)
Cu4—N4	1.934 (2)	C33—C38	1.419 (4)
Cu2—O11	1.986 (2)	C33—C31	1.466 (4)
Cu2—O2	1.9385 (17)	C33—C34	1.414 (4)
Cu2—O3	1.855 (2)	C38—C37	1.409 (4)
Cu2—N2	1.941 (2)	C14—C19	1.425 (4)
O11—N102	1.259 (3)	C14—C15	1.403 (4)
N102—O12	1.206 (3)	C31—C32	1.503 (4)
N102—O13	1.225 (3)	C6—C5	1.411 (4)
Cu6—O14B	2.45 (2)	C29—C30	1.523 (4)
Cu6—O8	1.9350 (18)	C29—C28	1.515 (4)
Cu6—O9	1.863 (2)	C52—C57	1.419 (4)
Cu6—O2W	2.0273 (19)	C52—C50	1.467 (4)
Cu6—N6	1.942 (2)	C52—C53	1.415 (4)
O14B—N202	1.28 (3)	C57—C56	1.414 (4)
N202—O16B	1.25 (2)	C50—C51	1.503 (4)
N202—O15B	1.26 (3)	C34—C35	1.371 (4)
N202—O16A	1.22 (2)	C44—C43	1.409 (4)
N202—O15A	1.196 (14)	C19—C18	1.419 (4)
N202—O14A	1.20 (2)	C37—C36	1.370 (4)
O2—C10	1.438 (3)	C40—C41	1.376 (4)
O5—C29	1.418 (3)	C21—C22	1.378 (4)
O8—C48	1.426 (3)	C35—C36	1.374 (5)
O4—C20	1.318 (3)	C56—C55	1.368 (5)
O1—C1	1.318 (3)	C5—C4	1.367 (5)
O7—C39	1.310 (3)	C53—C54	1.371 (5)
O9—C57	1.313 (3)	C2—C3	1.373 (4)
O3—C19	1.306 (4)	C18—C17	1.364 (5)
O6—C38	1.316 (3)	C4—C3	1.377 (5)
N5—C45	1.287 (4)	C17—C16	1.380 (6)
N5—C47	1.475 (3)	C55—C54	1.376 (5)
N1—C7	1.295 (3)	C24—C23	1.369 (5)
N1—C9	1.468 (3)	C43—C42	1.368 (6)
N3—C26	1.286 (3)	C15—C16	1.369 (5)
N3—C28	1.474 (3)	C22—C23	1.378 (5)
N4—C31	1.294 (3)	C41—C42	1.381 (6)
N4—C30	1.469 (3)	O1E—C2E	1.426 (6)
N2—C11	1.468 (3)	C2E—C3E	1.456 (8)
N2—C12	1.295 (4)	O4E—C5E	1.348 (6)
N6—C50	1.294 (3)	C5E—C6E	1.461 (8)
N6—C49	1.472 (4)	O7E—C8E	1.473 (9)
C7—C6	1.463 (4)	C8E—C9E	1.420 (4)
			
O10—Cu3—O7	72.48 (7)	C12—N2—C11	120.5 (2)
O5—Cu3—O10	95.79 (8)	C50—N6—Cu6	127.0 (2)
O5—Cu3—O7	91.62 (7)	C50—N6—C49	121.3 (2)
O5—Cu3—N3	85.86 (8)	C49—N6—Cu6	110.64 (16)
O4—Cu3—O10	84.02 (8)	N1—C7—C6	120.7 (2)
O4—Cu3—O5	170.39 (8)	N1—C7—C8	120.6 (3)
O4—Cu3—O7	97.46 (7)	C6—C7—C8	118.7 (2)
O4—Cu3—N3	92.84 (8)	O4—C20—C25	124.2 (2)
N3—Cu3—O10	170.79 (8)	O4—C20—C21	117.6 (2)
N3—Cu3—O7	116.59 (8)	C21—C20—C25	118.2 (2)
O10—Cu1—O4	72.34 (6)	O1—C1—C6	124.2 (2)
O2—Cu1—O10	92.40 (8)	O1—C1—C2	117.4 (3)
O2—Cu1—O4	93.94 (7)	C2—C1—C6	118.4 (2)
O2—Cu1—N1	86.00 (8)	N3—C26—C25	121.4 (2)
O1—Cu1—O10	86.17 (8)	N3—C26—C27	120.4 (2)
O1—Cu1—O2	170.60 (8)	C25—C26—C27	118.2 (2)
O1—Cu1—O4	94.47 (8)	O2—C10—C11	108.7 (2)
O1—Cu1—N1	93.42 (8)	O2—C10—C9	108.7 (2)
N1—Cu1—O10	167.74 (8)	C11—C10—C9	111.4 (2)
N1—Cu1—O4	119.88 (8)	C20—C25—C26	123.0 (2)
O10—Cu5—O1	72.65 (7)	C24—C25—C20	117.7 (3)
O8—Cu5—O10	94.68 (7)	C24—C25—C26	119.3 (3)
O8—Cu5—O1	93.29 (7)	O8—C48—C49	108.9 (2)
O8—Cu5—N5	86.72 (8)	O8—C48—C47	109.7 (2)
O7—Cu5—O10	84.42 (7)	C47—C48—C49	112.2 (2)
O7—Cu5—O8	171.86 (8)	N2—C11—C10	108.1 (2)
O7—Cu5—O1	94.15 (8)	N5—C45—C44	120.5 (2)
O7—Cu5—N5	92.98 (8)	N5—C45—C46	120.9 (3)
N5—Cu5—O10	171.32 (9)	C44—C45—C46	118.6 (3)
N5—Cu5—O1	115.87 (8)	O7—C39—C44	123.9 (3)
O5—Cu4—O1W	87.84 (8)	O7—C39—C40	117.7 (2)
O5—Cu4—N4	86.56 (8)	C40—C39—C44	118.4 (2)
O6—Cu4—O5	173.69 (9)	N2—C12—C14	121.1 (3)
O6—Cu4—O1W	90.97 (9)	N2—C12—C13	119.9 (3)
O6—Cu4—N4	95.34 (9)	C14—C12—C13	119.0 (3)
N4—Cu4—O1W	171.10 (9)	C38—C33—C31	123.9 (2)
O2—Cu2—O11	88.40 (8)	C34—C33—C38	117.4 (2)
O2—Cu2—N2	86.37 (8)	C34—C33—C31	118.7 (2)
O3—Cu2—O11	90.92 (9)	O6—C38—C33	125.7 (2)
O3—Cu2—O2	171.60 (8)	O6—C38—C37	116.1 (3)
O3—Cu2—N2	94.27 (9)	C37—C38—C33	118.1 (2)
N2—Cu2—O11	174.77 (9)	C19—C14—C12	122.9 (3)
N102—O11—Cu2	118.74 (17)	C15—C14—C12	119.4 (3)
O12—N102—O11	120.6 (3)	C15—C14—C19	117.7 (3)
O12—N102—O13	123.8 (3)	N4—C31—C33	121.2 (2)
O13—N102—O11	115.7 (2)	N4—C31—C32	120.4 (3)
O8—Cu6—O14B	91.3 (6)	C33—C31—C32	118.5 (2)
O8—Cu6—O2W	90.96 (8)	C1—C6—C7	123.7 (2)
O8—Cu6—N6	86.12 (9)	C5—C6—C7	118.7 (3)
O9—Cu6—O14B	97.2 (6)	C5—C6—C1	117.6 (3)
O9—Cu6—O8	171.27 (9)	O5—C29—C30	108.9 (2)
O9—Cu6—O2W	87.48 (9)	O5—C29—C28	108.9 (2)
O9—Cu6—N6	94.66 (9)	C28—C29—C30	112.4 (2)
O2W—Cu6—O14B	87.7 (8)	C57—C52—C50	123.6 (2)
N6—Cu6—O14B	97.4 (8)	C53—C52—C57	117.5 (3)
N6—Cu6—O2W	174.23 (9)	C53—C52—C50	119.0 (3)
N202—O14B—Cu6	122.5 (12)	O9—C57—C52	125.2 (3)
O16B—N202—O14B	113.4 (19)	O9—C57—C56	116.4 (3)
O16B—N202—O15B	117.3 (19)	C56—C57—C52	118.4 (3)
O15B—N202—O14B	123.7 (19)	N6—C50—C52	121.0 (2)
O15A—N202—O16A	114 (2)	N6—C50—C51	120.1 (3)
O15A—N202—O14A	119.3 (16)	C52—C50—C51	118.9 (3)
O14A—N202—O16A	125 (2)	N4—C30—C29	108.1 (2)
Cu3—O10—Cu1	106.62 (9)	N3—C28—C29	107.97 (19)
Cu5—O10—Cu3	106.11 (8)	N6—C49—C48	107.0 (2)
Cu5—O10—Cu1	105.99 (9)	N5—C47—C48	107.5 (2)
Cu2—O2—Cu1	115.44 (9)	C35—C34—C33	123.0 (3)
C10—O2—Cu1	107.68 (14)	N1—C9—C10	107.8 (2)
C10—O2—Cu2	106.34 (14)	C39—C44—C45	123.6 (2)
Cu4—O5—Cu3	119.32 (8)	C43—C44—C45	119.2 (3)
C29—O5—Cu3	109.95 (15)	C43—C44—C39	117.2 (3)
C29—O5—Cu4	106.28 (14)	O3—C19—C14	125.6 (2)
Cu6—O8—Cu5	120.77 (9)	O3—C19—C18	116.2 (3)
C48—O8—Cu5	108.23 (15)	C18—C19—C14	118.1 (3)
C48—O8—Cu6	106.02 (15)	C36—C37—C38	122.0 (3)
Cu3—O4—Cu1	96.50 (7)	C41—C40—C39	122.0 (3)
C20—O4—Cu3	124.71 (16)	C22—C21—C20	121.5 (3)
C20—O4—Cu1	137.80 (16)	C34—C35—C36	118.9 (3)
Cu1—O1—Cu5	93.56 (7)	C55—C56—C57	121.8 (3)
C1—O1—Cu1	125.52 (17)	C4—C5—C6	122.4 (3)
C1—O1—Cu5	140.83 (16)	C37—C36—C35	120.6 (3)
Cu5—O7—Cu3	96.20 (7)	C54—C53—C52	122.5 (3)
C39—O7—Cu3	138.45 (17)	C3—C2—C1	121.5 (3)
C39—O7—Cu5	124.21 (16)	C17—C18—C19	121.6 (3)
C57—O9—Cu6	125.90 (19)	C5—C4—C3	119.5 (3)
C19—O3—Cu2	126.46 (18)	C18—C17—C16	120.3 (3)
C38—O6—Cu4	125.81 (18)	C56—C55—C54	120.3 (3)
C45—N5—Cu5	128.00 (19)	C23—C24—C25	123.1 (3)
C45—N5—C47	121.5 (2)	C2—C3—C4	120.6 (3)
C47—N5—Cu5	109.65 (17)	C42—C43—C44	123.1 (3)
C7—N1—Cu1	127.63 (19)	C16—C15—C14	122.6 (3)
C7—N1—C9	120.5 (2)	C23—C22—C21	120.6 (3)
C9—N1—Cu1	110.30 (16)	C15—C16—C17	119.7 (3)
C26—N3—Cu3	127.41 (18)	C40—C41—C42	120.0 (3)
C26—N3—C28	121.8 (2)	C53—C54—C55	119.6 (3)
C28—N3—Cu3	109.53 (16)	C43—C42—C41	119.3 (3)
C31—N4—Cu4	127.25 (19)	C24—C23—C22	118.9 (3)
C31—N4—C30	121.8 (2)	O1E—C2E—C3E	106.6 (5)
C30—N4—Cu4	110.24 (16)	O4E—C5E—C6E	110.7 (5)
C11—N2—Cu2	110.48 (17)	C9E—C8E—O7E	104.3 (9)
C12—N2—Cu2	127.20 (19)		
			
Cu3—O5—C29—C30	−83.1 (2)	N2—Cu2—O3—C19	5.5 (2)
Cu3—O5—C29—C28	39.9 (2)	N2—C12—C14—C19	12.4 (4)
Cu3—O4—C20—C25	−25.9 (3)	N2—C12—C14—C15	−165.9 (3)
Cu3—O4—C20—C21	155.9 (2)	N6—Cu6—O9—C57	9.4 (2)
Cu3—O7—C39—C44	167.82 (19)	C7—N1—C9—C10	−165.0 (2)
Cu3—O7—C39—C40	−10.6 (4)	C7—C6—C5—C4	178.6 (3)
Cu3—N3—C26—C25	−13.9 (4)	C20—C25—C24—C23	−0.1 (5)
Cu3—N3—C26—C27	165.9 (2)	C20—C21—C22—C23	−0.2 (6)
Cu3—N3—C28—C29	30.0 (3)	C1—C6—C5—C4	−1.5 (4)
Cu1—O2—C10—C11	−78.1 (2)	C1—C2—C3—C4	−0.4 (5)
Cu1—O2—C10—C9	43.3 (2)	C26—N3—C28—C29	−162.2 (2)
Cu1—O4—C20—C25	168.43 (19)	C26—C25—C24—C23	180.0 (4)
Cu1—O4—C20—C21	−9.8 (4)	C25—C20—C21—C22	−1.2 (5)
Cu1—O1—C1—C6	−23.0 (4)	C25—C24—C23—C22	−1.3 (7)
Cu1—O1—C1—C2	159.2 (2)	C11—N2—C12—C14	179.0 (2)
Cu1—N1—C7—C6	−15.7 (3)	C11—N2—C12—C13	−1.6 (4)
Cu1—N1—C7—C8	164.7 (2)	C11—C10—C9—N1	72.4 (3)
Cu1—N1—C9—C10	28.2 (3)	C45—N5—C47—C48	−159.8 (2)
Cu5—O8—C48—C49	−82.9 (2)	C45—C44—C43—C42	177.0 (4)
Cu5—O8—C48—C47	40.2 (2)	C39—C44—C43—C42	−1.1 (6)
Cu5—O1—C1—C6	152.5 (2)	C39—C40—C41—C42	−0.6 (6)
Cu5—O1—C1—C2	−25.3 (4)	C12—N2—C11—C10	−171.9 (2)
Cu5—O7—C39—C44	−27.5 (4)	C12—C14—C19—O3	2.8 (4)
Cu5—O7—C39—C40	154.0 (2)	C12—C14—C19—C18	−178.7 (3)
Cu5—N5—C45—C44	−13.1 (4)	C12—C14—C15—C16	178.2 (3)
Cu5—N5—C45—C46	168.1 (2)	C33—C38—C37—C36	−0.5 (4)
Cu5—N5—C47—C48	29.9 (3)	C33—C34—C35—C36	−0.1 (5)
Cu4—O5—C29—C30	47.4 (2)	C38—C33—C31—N4	4.6 (4)
Cu4—O5—C29—C28	170.32 (17)	C38—C33—C31—C32	−174.6 (3)
Cu4—O6—C38—C33	−1.1 (4)	C38—C33—C34—C35	0.1 (4)
Cu4—O6—C38—C37	179.6 (2)	C38—C37—C36—C35	0.5 (5)
Cu4—N4—C31—C33	−10.9 (4)	C14—C19—C18—C17	0.5 (4)
Cu4—N4—C31—C32	168.2 (2)	C14—C15—C16—C17	0.6 (6)
Cu4—N4—C30—C29	17.8 (2)	C31—N4—C30—C29	−171.1 (2)
Cu2—O11—N102—O12	17.8 (4)	C31—C33—C38—O6	1.8 (4)
Cu2—O11—N102—O13	−162.3 (3)	C31—C33—C38—C37	−178.9 (3)
Cu2—O2—C10—C11	46.2 (2)	C31—C33—C34—C35	179.3 (3)
Cu2—O2—C10—C9	167.61 (17)	C6—C1—C2—C3	−1.8 (5)
Cu2—O3—C19—C14	−11.2 (4)	C6—C5—C4—C3	−0.6 (5)
Cu2—O3—C19—C18	170.3 (2)	C52—C57—C56—C55	2.2 (5)
Cu2—N2—C11—C10	22.4 (2)	C52—C53—C54—C55	−0.3 (6)
Cu2—N2—C12—C14	−17.9 (4)	C57—C52—C50—N6	14.5 (4)
Cu2—N2—C12—C13	161.5 (2)	C57—C52—C50—C51	−164.8 (3)
O11—Cu2—O3—C19	−175.2 (2)	C57—C52—C53—C54	0.7 (5)
Cu6—O14B—N202—O16B	−108 (3)	C57—C56—C55—C54	−1.9 (6)
Cu6—O14B—N202—O15B	99 (4)	C50—N6—C49—C48	−168.5 (2)
Cu6—O8—C48—C49	48.0 (2)	C50—C52—C57—O9	0.9 (4)
Cu6—O8—C48—C47	171.09 (17)	C50—C52—C57—C56	178.9 (3)
Cu6—O9—C57—C52	−13.1 (4)	C50—C52—C53—C54	−179.8 (3)
Cu6—O9—C57—C56	168.9 (2)	C30—N4—C31—C33	179.5 (2)
Cu6—N6—C50—C52	−16.4 (4)	C30—N4—C31—C32	−1.4 (4)
Cu6—N6—C50—C51	162.9 (2)	C30—C29—C28—N3	74.9 (3)
Cu6—N6—C49—C48	22.4 (2)	C28—N3—C26—C25	−179.5 (2)
O14B—Cu6—O9—C57	−88.6 (8)	C28—N3—C26—C27	0.4 (4)
O10—Cu3—O4—Cu1	5.17 (7)	C28—C29—C30—N4	−163.9 (2)
O10—Cu3—O4—C20	−165.2 (2)	C49—N6—C50—C52	176.4 (2)
O10—Cu1—O1—Cu5	8.93 (7)	C49—N6—C50—C51	−4.3 (4)
O10—Cu1—O1—C1	−173.9 (2)	C49—C48—C47—N5	74.7 (3)
O10—Cu5—O7—Cu3	6.37 (8)	C47—N5—C45—C44	178.6 (2)
O10—Cu5—O7—C39	−163.5 (2)	C47—N5—C45—C46	−0.3 (4)
O2—C10—C11—N2	−45.5 (3)	C47—C48—C49—N6	−168.3 (2)
O2—C10—C9—N1	−47.3 (3)	C34—C33—C38—O6	−179.1 (3)
O5—C29—C30—N4	−43.1 (3)	C34—C33—C38—C37	0.2 (4)
O5—C29—C28—N3	−45.9 (3)	C34—C33—C31—N4	−174.5 (3)
O8—C48—C49—N6	−46.7 (3)	C34—C33—C31—C32	6.3 (4)
O8—C48—C47—N5	−46.5 (3)	C34—C35—C36—C37	−0.2 (5)
O4—Cu1—O1—Cu5	80.85 (7)	C9—N1—C7—C6	180.0 (2)
O4—Cu1—O1—C1	−102.0 (2)	C9—N1—C7—C8	0.4 (4)
O4—C20—C25—C26	3.0 (4)	C9—C10—C11—N2	−165.2 (2)
O4—C20—C25—C24	−176.9 (3)	C44—C39—C40—C41	0.3 (5)
O4—C20—C21—C22	177.1 (3)	C44—C43—C42—C41	0.8 (7)
O1—Cu5—O7—Cu3	78.46 (7)	C19—C14—C15—C16	−0.1 (5)
O1—Cu5—O7—C39	−91.4 (2)	C19—C18—C17—C16	0.0 (5)
O1—C1—C6—C7	4.7 (4)	C40—C39—C44—C45	−177.4 (3)
O1—C1—C6—C5	−175.2 (2)	C40—C39—C44—C43	0.5 (4)
O1—C1—C2—C3	176.2 (3)	C40—C41—C42—C43	0.1 (7)
O7—Cu3—O4—Cu1	76.57 (7)	C21—C20—C25—C26	−178.8 (3)
O7—Cu3—O4—C20	−93.8 (2)	C21—C20—C25—C24	1.3 (4)
O7—C39—C44—C45	4.1 (4)	C21—C22—C23—C24	1.4 (7)
O7—C39—C44—C43	−177.9 (3)	C27—C26—C25—C20	−162.0 (3)
O7—C39—C40—C41	178.9 (3)	C27—C26—C25—C24	18.0 (4)
O9—C57—C56—C55	−179.6 (3)	C56—C55—C54—C53	0.8 (6)
O3—C19—C18—C17	179.1 (3)	C5—C4—C3—C2	1.6 (5)
O2W—Cu6—O9—C57	−175.9 (2)	C8—C7—C6—C1	−165.0 (3)
O6—C38—C37—C36	178.8 (3)	C8—C7—C6—C5	14.9 (4)
O1W—Cu4—O6—C38	−177.0 (2)	C53—C52—C57—O9	−179.6 (3)
N5—Cu5—O7—Cu3	−165.33 (8)	C53—C52—C57—C56	−1.6 (4)
N5—Cu5—O7—C39	24.8 (2)	C53—C52—C50—N6	−165.0 (3)
N5—C45—C44—C39	17.2 (4)	C53—C52—C50—C51	15.7 (4)
N5—C45—C44—C43	−160.8 (3)	C2—C1—C6—C7	−177.5 (3)
N1—Cu1—O1—Cu5	−158.80 (8)	C2—C1—C6—C5	2.6 (4)
N1—Cu1—O1—C1	18.4 (2)	C18—C17—C16—C15	−0.5 (6)
N1—C7—C6—C1	15.4 (4)	C13—C12—C14—C19	−167.0 (3)
N1—C7—C6—C5	−164.7 (2)	C13—C12—C14—C15	14.7 (4)
N3—Cu3—O4—Cu1	−166.14 (8)	C46—C45—C44—C39	−164.0 (3)
N3—Cu3—O4—C20	23.5 (2)	C46—C45—C44—C43	18.1 (4)
N3—C26—C25—C20	17.9 (4)	C15—C14—C19—O3	−178.9 (3)
N3—C26—C25—C24	−162.2 (3)	C15—C14—C19—C18	−0.4 (4)
N4—Cu4—O6—C38	−3.3 (2)		

### Hydrogen-bond geometry (Å, º)

**Table:**

*D*—H···*A*	*D*—H	H···*A*	*D*···*A*	*D*—H···*A*
O10—H10···O11	0.51 (4)	2.43 (4)	2.854 (3)	142 (6)
O10—H10···O2*W*	0.51 (4)	2.75 (3)	2.910 (3)	103 (4)
O10—H10···O1*W*	0.51 (4)	2.73 (4)	3.155 (3)	143 (6)
O1*W*—H1*WA*···O1*E*	0.86	2.26	2.625 (4)	106
O1*W*—H1*WB*···O13	0.86	2.21	2.876 (4)	135
C11—H11*B*···O4*E*^i^	0.97	2.31	3.280 (4)	174
C18—H18···O13^ii^	0.93	2.54	3.328 (4)	143
O4*E*—H4*E*···N202	0.82	2.66	3.447 (5)	161
O4*E*—H4*E*···O16*B*	0.82	2.45	3.13 (2)	141
O4*E*—H4*E*···O15*B*	0.82	2.12	2.87 (3)	151

Symmetry codes: (i) −*x*+1, −*y*+1, −*z*+1; (ii) −*x*+1, −*y*+1, −*z*.
